# Clean Label Approaches in Cheese Production: Where Are We?

**DOI:** 10.3390/foods14050805

**Published:** 2025-02-26

**Authors:** Jaime Fernandes, Sandra Gomes, Fernando H. Reboredo, Manuela E. Pintado, Olga Amaral, João Dias, Nuno Alvarenga

**Affiliations:** 1 UTI—Unidade de Tecnologia e Inovação, Instituto Nacional de Investigação Agrária e Veterinária IP, Quinta do Marquês, 2780-157 Oeiras, Portugal; 2 NOVA School of Science and Technology, Universidade Nova de Lisboa, 2829-516 Caparica, Portugal; 3 GeoBioTec Research Center, NOVA School of Science and Technology, Universidade Nova de Lisboa, 2829-516 Caparica, Portugal; 4 CBQF—Centro de Biotecnologia e Química Fina, Laboratório Associado, Escola Superior de Biotecnologia, Universidade Católica Portuguesa, Rua Diogo Botelho 1327, 4169-005 Porto, Portugal; 5 School of Agriculture, Polytechnic University of Beja, Rua Pedro Soares, 7800-295 Beja, Portugal; 6 MED—Mediterranean Institute for Agriculture, Environment and Development, University of Évora, 7006-554 Évora, Portugal

**Keywords:** clean label, cheese, dairy industry, non-thermal processing, microbiological control, edible coatings, plant extracts, food safety

## Abstract

The Clean Label concept has gained significant traction in the cheese industry due to consumer preferences for minimally processed cheeses free from synthetic additives. This review explores different approaches for applying Clean Label principles to the cheese industry while maintaining food safety, sensory quality, and shelf life. Non-thermal technologies, such as high-pressure processing (HPP), pulsed electric fields (PEF), ultra-violet (UV), and visible light (VL), are among the most promising methods that effectively control microbial growth while preserving the nutritional and functional properties of cheese. Protective cultures, postbiotics, and bacteriophages represent microbiological strategies that are natural alternatives to conventional preservatives. Another efficient approach involves plant extracts, which contribute to microbial control, and enhance cheese functionality and potential health benefits. Edible coatings, either alone or combined with other methods, also show promising applications. Despite these advantages, several challenges persist: higher costs of production and technical limitations, possible shorter shelf-life, and regulatory challenges, such as the absence of standardized Clean Label definitions and compliance complexities. Further research is needed to develop and refine Clean Label formulations, especially regarding bioactive peptides, sustainable packaging, and advanced microbial control techniques. Addressing these challenges will be essential for expanding Clean Label cheese availability while ensuring product quality and maintaining consumer acceptance.

## 1. Introduction

### 1.1. Clean Label: What Is It?

The end of the 19th century marks the beginning of large-scale industrialization of the agri-food industry [1]. The beginning of the food industry revolution was focused on food preservation and shelf-life. While this evolution led to an interest in food safety (1920–1930s), it was only after the Second World War that greater concern with the nutritional quality of food began to emerge [2], leading to the creation of a whole set of conservation methodologies, not only physical (pasteurization, freezing, refrigeration, and others) but also chemical, using additives and preservatives that met a set of market demands and a context of demographic, social, and cultural changes [3].

Since the 1970s, the presence of additives and preservatives in food has been perceived negatively by consumers. This gave rise to what Haen [4] termed “the paradox of E-numbers”, where the “E” represents chemicals additives used for food safety, yet consumers identified them as harmful “chemicals in food products”. As a result, more and more consumers have been looking to find foods free of these additives that they consider unnatural, artificial, and unhealthy [5]. This negative perception is also associated with food products, animal or vegetable, of unethical origin or that do not respect animal welfare [6]. Thus, a counter-current movement emerged that was called Clean Label.

The definition of Clean Label is complex, but it can go from the most simplistic definition, given by Michael Pollan, who says that consumers should not eat foods that: “have more than 5 ingredients or ingredients that they cannot pronounce” [7]. The evolution and development of this concept gave rise to more concrete definitions: “[Clean Label products] are free of chemical additives, with an easy-to-understand list of ingredients” and introduces the concept of traditional production by referring to the fact that “production [of these foods] must follow traditional techniques with limited processing” [8]. It is also suggested that the origin of the ingredients can be called “organic” as a way of reinforcing the non-use of foreign chemicals in their production or processing [9]. The absence of legislation in this field still renders its definition and characterization somewhat ambiguous and open to interpretation [10].

In summary, the Clean Label has emerged as a result of consumer choice and demand, which seeks simpler and more natural food and with a consumption impact that is, if not positive, at least neutral on their health and that of their family [11]. The demand for this category of products and foods has been met by retailers and producers, with a global market of 47 billion US dollars in 2022 [12].

### 1.2. Clean Label Applied to Cheese

Cheese has been part of the human diet for about eight thousand years, obtained from the coagulation of milk and later fermentation of curd. Current data suggests that its production was a way of transforming milk, potentially toxic to adult prehistoric Man, who was unable to digest lactose, into an edible food with low levels of lactose, thus making cheese a nutritionally significant food [13]. Since then, cheese has evolved in shape, colors, and flavors as a reflection of different latitudes, cultures, production processes, and consumer demands [14]. The conservation of cheeses, from production until reaching the consumer, has been a challenge for the application of Clean Label [15]. In hard and soft cheese, undesirable microbial development, such as surface molds, leads to a loss of product value with economic losses for the producer and retailers [16,17]. To prevent the development of these molds, biocides are applied, such as natamycin, nisin, nitrates, sorbates, and phosphates. These products authorized by the U.S. Food and Drug Administration (FDA) and the European Food Safety Authority (EFSA), and are considered safe for the consumer [18,19], but, more and more, the consumer has the desire to avoid eating food that contains synthetic preservatives, or whose origin is not as natural as possible [20].

The market and producers have been trying to meet this expectation from the consumer. Retail companies specializing in products of natural or organic origin were the first to try to implement Clean Label on cheeses, as is the case of the giant Whole Foods Market, Inc. This company only allows the use of natamycin in cheeses that are covered with wax, that is, in cases where the biocide is not in direct contact with the edible parts of the product being sold [21].

Nevertheless, manufacturers must ensure that the products made available to the consumer are safe, comply with standards and legislation, and still maintain the organoleptic and sensory characteristics that the consumer is used to, and expects and desires from the dairy product they consume.

This review aims to analyze these emerging technologies while also considering advancements in more traditional methods to understand the current state of Clean Label implementation in the cheese industry.

## 2. Methodology

A systematic review was adopted consisting of a targeted search in Google Scholar, ScienceOpen, and Scopus electronic databases for terms related to Clean Label in the cheese and dairy sector, new trends in Clean Label, and emergent food technology specifically focusing on dairy products. Papers published in the last five years (2019–2024) were considered, except when the topic in question did not have enough articles published in that time frame. The research took place between August and December 2024.

## 3. Physical Approaches

Although the subject of this article is the application of clean labelling to cheeses as end products, it is essential to remember that much of the microbiological contamination of cheeses comes from the milk. Reducing the microbial contents in milk is fundamental to removing a whole range of pathogenic elements and microbiological contaminants from the production process.

The main approaches used in the dairy industry are physical methods that reduce microbial presence through physical processes such as temperature, electric fields, radiation, and modified atmosphere.

### 3.1. Thermal

In the dairy industry, heat treatments are the most important, oldest, and most generally used processes for reducing microbial content and ensuring consumer safety.

#### 3.1.1. Pasteurization

Pasteurization is the most widely used process for ensuring the safety of the dairy products in the market. However, this process also results in the loss of milk’s constituent compounds, both nutritionally (reduction of vitamins and proteins with biological value) and from a technological (reduction in micelar calcium content) point of view [22,23]. Although these reductions can be compensated with subsequent additions, such as the addition of calcium for cheese production, this procedure tends to go against the idea of Clean Label in terms of the intrusion of foreign elements into the matrix.

But even in this field there has been technological innovation such as new methods promoting faster, more localized, and more efficient heating.

#### 3.1.2. Induced Electric Field

One method that has already been used in other foods, such as juices, is the Induced Electric Field (IEF), which has begun to be used in milk. IEF is based in the application of an oscillating magnetic field, produced by an electromagnetic effect, which leads to an increase in temperature over a much shorter period and in a much smaller flow section. This process, seen as a combined thermal and non-thermal action [24,25], subjects the milk to an electromagnetic field that destabilizes the cell membranes and destroys the microorganisms present in the milk [26,27]. The results have been promising with the reduction or even total elimination of microorganisms with pathogenic potential such as *Escherichia coli*, *Staphylococcus aureus* and *Salmonella*, without alterations to the milk’s nutritional profile such as a reduction in protein or lactoferrin [26,28].

This process has presented some challenges for application at large-scale industrial level and for evaluating energy efficiency [24,25].

#### 3.1.3. Freezing

Since the massification of freezing processes for food preservation, the impact on cheese storage has been studied. This is a process that does not lead to the reduction or alteration of the microorganisms present in cheese, but can significantly reduce their development, making it possible to store cheese when there is an increase in production and it is not immediately available for distribution chains, a situation relevant for cheeses with seasonal production [29].

One of the main problems caused by negative temperature in cheese is directly related to its structure. During freezing, ice crystallization induces casein dehydration and the development of concentration gradients within the cheese matrix, leading to undesirable alterations in texture and functional properties [30]. These changes lead to the cheese losing its characteristics and therefore its acceptance by the consumer, rather than changes in its nutritional composition [31].

To overcome these changes, the best freezing procedures have been studied. The most recent study by Pax et al. [32] on the freezing of Mozzarella cheese blocks highlights on the best methods for the freezing process: a slow freezing process, lasting approximately 114 h, and a thawing process. This process was found to restore the structure and functionality of cheese that had been frozen at −18 °C for six months.

However, this procedure is highly specific to each type of cheese and can be both time consuming and financially burdensome processes, in addition to the significant electricity consumption required for freezing and thawing. All these factors must be carefully considered to ensure that the product is not only Clean Label but also sustainable [29].

### 3.2. Non-Thermal

The non-thermal approach, which does not increase significantly the temperature of the milk and thus preserves its nutritional and technological characteristics, has been widely studied both for reducing the microbiological load of milk and for direct application to cheese, either in the final product or during manufacturing.

The most emerging technological processes that can be more directly associated with Clean Label products are high pressure processing, pulsed light, ultraviolet treatment, pulsed electric fields, and ozone application.

#### 3.2.1. High Pressure Processing (HPP)

HPP is an advanced non-thermal food preservation technology that ensures safety and quality. Applying ultra-high hydrostatic pressure up to a level between 100 to 1000 MPa damages microbial cell membranes and inactivates enzymes, keeping bacterial growth from happening while retaining nutritional and sensory food characteristics. HPP has been recognized as a technology that ensures a longer shelf life and safety for food that can be consumed without the need for thermic processes, making it a valuable asset for the food industry and has proven to be effective in reducing many pathogenic microorganisms present in milk. Table 1 summarizes the most recent studies on this subject [33,34,35].

The data shows that the effectiveness of HPP in reducing microorganisms is highly dependent on the pressure and processing time parameters used, as observed with *Escherichia coli*. Additionally, even slight variations in these parameters appear to influence the effects of HPP: *Staphylococcus aureus* 765 exhibited a reduction of more than 8.0 log CFU·mL^−1^, nearly double the reduction observed in another study where the reduction was 4.70 log CFU·mL^−1^ [39].

It should also be noted that, in addition to affecting pathogens, HPP also impacts on bacteria essential for cheese production, especially those produced from raw milk and which benefit from the milk’s native bacterial flora, such as lactic acid bacteria (LAB).

Without affecting the sensory qualities of the milk [36,37], HPP changes some parameters such as the size of the casein micelles. When the treatment is carried out at a pressure >300 MPa, there is a 50% decrease in casein micelles size. At pressures between 200 and 300 MPa there is a decrease of 20 to 30%, but this change is reversible during milk storage. Below 200 MPa, there are no changes in the size of the casein micelles. This parameter is particularly relevant for cheese production [40].

A study on the effect of milk treatment on the quality of traditional cheeses, such as *Serra da Estrela* cheese made from raw sheep’s milk, demonstrated that after being subjected to 600 MPa for 6 min, there were no sensory or textural changes, nor any significant alterations in physicochemical parameters [41]. However, Serna-Hernandez et al. [40] reported that the use of milk treated with HPP results in some changes in the cheese production process, including modifications in coagulation time, acceleration of ripening, increased production yields, and changes in the physicochemical and sensory properties.

The application of HPP to cheeses has been used to alter the ripening times, stopping the ripening process, eliminating the microorganisms responsible for this process, and reducing bitterness in overripened cheeses [42]. Nuñez et al. [43] stated that HPP causes changes in both internal and external color of cheeses subjected to HPP, but these changes depend not only on the parameters used in HPP but also on the type of cheese.

The contradictory results of the studies highlight the steps still needed in the field of HPP, such as the standardization of application parameters. This has led EFSA to state that “Current data are not robust enough to support the proposal of an appropriate indicator to verify the efficacy of HPP under the current HPP conditions applied by the industry” [44]. Furthermore, this process has been carried out in batches, and there are limitations to industrial application in continuous production.

#### 3.2.2. Pulsed Light (PL), Ultraviolet (UV), and Visible Light (VL)

PL is a non-thermal treatment where a flash of light is produced during a high-voltage discharge applied to an ampoule containing an inert gas (usually xenon). It is an energetic pulse of short duration, less than 1 s, but of high power that covers a wavelength of between 200 and 1000 nm [45,46]. PL has demonstrated potential for inhibiting microorganisms and, due to its characteristics, is applicable to the surface of food. This enables it to preserve the food’s quality while also being suitable for use in packaged products [47,48].

In the cheese industry, the use of PL has been studied in cheeses whose characteristics preclude the use of other preservative substances, such as fresh cheeses.

Checking the data for PL in Table 2 shows its effect against *Enterobacteriaceae*, *Pseudomonas* spp. and *Listeria* spp. in both fresh [46,49] and ripened cheeses [45,50].

The research carried out by Fernández et al. [50] found immediate changes in the odor and flavor of the samples with increase on sulphur notes, although, these notes disappeared with cold storage, resulting in a final product without any alteration regarding sensory characteristic or significant physicochemical changes.

The use of light in UV range, both in the treatment of milk and in the cheese itself after production, has been studied in the context of Clean Label and its perception among consumers [55,56]. The data in Table 2 regarding UV light demonstrate its effectiveness against *Escherichia coli* O157:H7, *Listeria monocytogenes, Salmonella typhimurium* and *Staphylococcus aureus* [52,53]. Ricciardi et al. [46] demonstrated effective changes with the production of small peptides capable of interacting with lipids and carbohydrates, thus producing sets of aggregates that alter the protein interaction of the cheese in an external layer of 1.5 mm equivalent to the capacity penetrative UV-C (λ = 200–300 nm), radiation that has been individually studied and evaluated in its applicability to the cheese industry. However, other research has reported that no change was detected in color, texture or sensory analysis [53].

More recent studies have used Light-Emitting Diodes (LEDs) instead of low-pressure mercury lamps, offering a simpler and more economical method with lower energy costs. For example, Oliveira et al. [57], using UVC diodes applied to water, at wavelength of 265 nm, achieved 3 log, 1 log and 5 log inactivation of *Aspergillus fumigatus*, *Aspergillus niger* and *Aspergillus terreus*, respectively.

The application of UV treatments to milk, in addition to reducing the microbial load, increases Vitamin D_3_ levels by converting 7-dehydricholesterol into this vitamin [58], providing a significant benefit in populations with a deficiency in this vitamin [59].

The application of UV can have negative effects: in milk, it modifies the fatty acid profile, increases the presence of thiobarbituric acid and reactive substances, and can cause the precipitation of proteins, thus altering the nutritional characteristics of the product; in the case of cheese, changes in color, oxidation of proteins, a reduction in pH, an increase in the levels of aldehydes and hydrocarbons have been reported, leading to sensory changes, although it should be noted that these changes only occur in the areas of exposure, i.e., on the cover of the cheese, and do not significantly affect ripened cheeses [55].

As already mentioned, the development of LEDs has made it possible to generate electromagnetic radiation sources in the visible range (400 to 700 nm) that are cheaper, easier to install, and highly selective for the desired wavelengths. This development has made it possible to use VL to verify that certain wavelengths could more effectively control the proliferation of microorganisms.

The data compiled in Table 2 demonstrate the effectiveness of VL in control or elimination of microorganisms such as *Escherichia coli*, *Listeria monocytogenes*, *Pseudomonas aeruginosa Pseudomonas fluorescens*, *Salmonella typhimurium* and *Staphylococcus aureus* both in fresh, ripened, and packaged cheeses [54], as well as in milk, with no change in the constituents of the milk, with the exception of riboflavin (vitamin B_2_) due to its sensitivity to radiation at this wavelength [51].

Its application in milk processing industries and dairy farms is quite promising, since it is a user-friendly technology with no specialized training required or occupational reported hazards. Photoinactivation with VL is, therefore, particularly advantageous for the dairy industry compared to UV-C light-based inactivation. This is due to minimal penetration of UV-C light in light-scattering aqueous solutions, its nonspecific induction of oxidation of biological materials, and the potential hazards it poses to the eyes and skin of workers [51].

The use of PL, UV, and VL to control microbial contamination has been widely applied in connection with packaged products that are exposed to light after being sealed. This approach has proven to be highly effective, but its application must always consider the type and thickness of the coating to maximize its effectiveness [47,54,60].

Lastly, PL, UV, and VL can be used to disinfect spaces and surfaces, reducing the need for chemical detergents that could inadvertently enter the production chain and reach the consumer [58].

#### 3.2.3. Pulsed Electric Field (PEF)

PEF technology is one of the promising novel non-thermal methods with great potential to replace conventional pasteurization techniques. Generally, PEF is effective in inactivating pathogenic and spoilage microorganisms while preserving nutrients, enzymes, and other desirable sensory properties of food products such as texture and taste. It involves the processing of liquid foods, such as milk, by applying brief high-voltage electric pulses in batch or continuous flow systems with intensities of 20−80 kV·cm^−1^ for several microseconds [25,61,62,63].

Recent research has demonstrated the capability of PEF to ensure microbial safety and maintain nutritional quality while preserving enzymatic activity in milk [64]. The microbial inactivation by PEF is mainly achieved by electroporation, the disruption of cell membranes, or irreversible pore formation under extreme intensities of the electric field. Besides this, it may directly affect the cytoplasmic content of microorganisms and enhance its decontamination capabilities [65].

The application of PEF treatment led to a reduction of *Staphylococcus aureus* and *Pseudomonas putida* of 3.2 log and 4.8 log, respectively, while exposed to an electric field of 2.9 kV·cm^−1^ with a frequency of 100 Hz [66].

In another study regarding milk, it was demonstrated that PEF can significantly reduce bacterial counts in samples treated under the following conditions: PEF at 24 kV.cm^−1^, pulse duration 25 µs, 20 pulses, and a reduction of 2.43 log·mL^−1^ in coliforms and 0.9 log·mL^−1^ in the total bacterial count was observed [67].

Despite the breakthroughs in the field of PEF, challenges remain in scaling up from small-scale laboratory equipment to industrial scale. The process of uniformity and conditions are greatly different between lab-scale and commercial-scale equipment. Additionally, the higher capital cost of PEF compared to pasteurization justifies its application primarily to premium dairy products, such as liquid infant food, dairy bioactive products, fruit–dairy blends, or raw milk cheeses.

Further research is needed to address safety and health-related issues of PEF-treated dairy products to systematically meet regulatory requirements. Considering the drawbacks and critical control points in PEF processing is crucial when designing an efficient industrial-scale PEF system, given the specificity of dairy products when compared to other liquid foods. The combination of PEF with other preservation techniques may provide synergistic benefits, making it possible to use milk to produce cheese with a cleaner label approach [25,65].

#### 3.2.4. Ozone (O_3_)

The use of O_3_, a molecular gas made up of three oxygen atoms, has aroused interest among promoters of a total Clean Label in the cheese industry.

Studies on the application of ozone in milk, as a raw material for cheese production, have been promising. In addition to reducing the microbial component, as evidenced by Oliveira Souza et al. [68] in the inhibition of *Escherichia coli*, it also allows the degradation of the aflatoxin M1 by 18.9%.

In the cheese ripening and storage process, the use of O_3_ allowed the control of surface microbial development. Studies by Grasso et al. [69] revealed the effectiveness of the process of ripening pecorino cheese, with a gaseous ozone treatment at 200 ppb for 8 h a day (overnight) limiting the growth and significantly reducing bacteria, yeast, and mold counts starting from 75 days of ripening without altering the characteristics of the cheese and its acceptance in consumer testing.

However, ozone can lead to lipid oxidation, a free radical complex chain reaction due to its high lipid content, which is particularly susceptible to this oxidation. Evidence of such an occurrence has been observed, leading to changes in the chemical and rheological profile of cheese, as well as its acceptance among consumers [70]. Nonetheless, according to the same author: “Further studies are needed to identify the ideal concentration–time combinations so that, on the one hand, the antimicrobial effect of ozone can be exploited without incurring sensory defects due to its pro-oxidant action, and, on the other, its ability to modify the characteristics of some molecules to obtain products with specific functions”.

Ozone can be an effective approach to eliminate the use of chemical products in the disinfection processes of production facilities (pipes, surfaces, vats, etc.), particularly in the form of ozonated water, thereby reducing contamination of the raw material during the production process [70]. Marino et al. [71] studied the action of applying ozone on *Pseudomonas fluorescens*, *Staphylococcus aureus*, and *Listeria monocytogenes* using three treatment techniques: immersion in ozonized water with 0.5 ppm, which achieved a reduction after 20 min of between 1.61 and 2.14 log CFU·cm^−2^; ozonized water underflow resulting in a reduction of between 3.26 and 5.23 log CFU·cm^−2^; and the application of ozone in the gaseous form, with a concentration of between 0.2 and 20 ppm. At the lowest concentration (0.2 ppm), estimated inactivation of 2.01–2.46 log CFU·cm^−2^ was obtained after 60 min. Complete inactivation of *Listeria monocytogenes* was achieved with a higher concentration of O_3_ while managing to reduce *Pseudomonas fluorescens* to 5.51 log CFU·cm^−2^ (60 min) and *Staphylococcus aureus* to 4.72 log CFU·cm^−2^ (20 min).

#### 3.2.5. Filtration

Filtration, particularly microfiltration, is a non-thermal approach that uses membranes to separate microorganisms from milk, and can achieve a microbial reduction comparable to pasteurization, with studies showing a reduction of up to 3.7 log CFU·mL^−1^ in the microbial count [72,73,74]. The process successfully eliminates a wide range of microorganisms, including thermoduric and spore-forming bacteria, which are not always inactivated by pasteurization [75]. However, this method is not without its challenges: the increased viscosity of milk at lower temperatures requires greater mechanical energy, and the need for in-line cooling increases thermal energy consumption, affecting the process’s sustainability [75]. In addition, issues such as membrane fouling and the need for effective cleaning protocols can complicate the filtration process, potentially reducing its efficiency and increasing operating costs [72]. Despite these challenges, microfiltration remains a promising approach to improving the microbial safety of milk while maintaining its nutritional and sensory properties.

## 4. Botanical Approach

The use of plant-based options has been widely studied, and a substantial amount of literature has been produced on the topic. Plants can be applied in various ways: directly during the production or ripening process (e.g., thyme, rosemary, chestnut, gentian and vine leaves, etc.) in the most diverse forms (whole parts, pulverized, etc.) or through the extraction of compounds either via maceration, decoction, or, for those with more expertise, the use of essential oil (EO) or ethanolic extracts (EE) [76].

**Figure 1:**
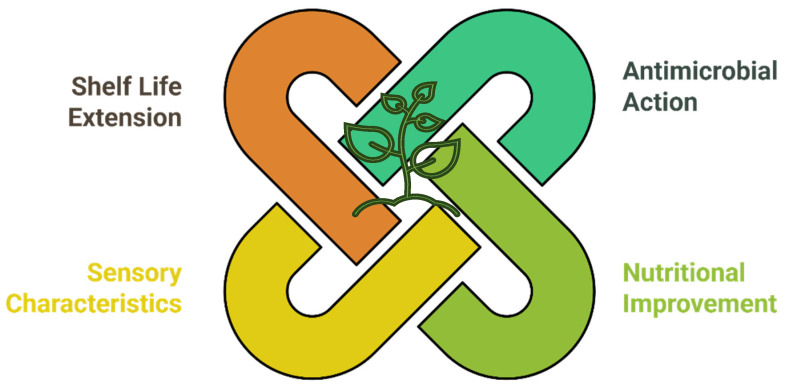
Potential synergies between botanical approaches in improving Clean Label: the integration of plant-based solutions can contribute to extending shelf life, antimicrobial action, nutritional improvement, and enhancing sensory characteristics.

The botanical approach covers four areas that can enable Clean Label in cheeses: antimicrobial action; nutritional improvement and functional properties; improvement of sensory characteristics; and increase in the product’s shelf life [77]. Figure 1 gives a schematic picture of the potential synergy of botanical approaches.

### 4.1. Antimicrobial Action

The active compounds obtained from plants are secondary metabolites and their function is often referred to as being compounds that enable the plant’s defense against not only higher predators, but also, because of their antimicrobial characteristics, they allow to control and reduce harmful microbial development in the cheese [78].

Table 3 shows a summary of some studies in which both EO and EE were used to prevent microbiological proliferation.

Oregano (*Origanum vulgare* L.) is one of the extracts upon which the most studies have been carried out in the last five years. Its inhibitory action has been seen in microorganisms that are problematic for cheese preservation, such as *Aspergillus flavus*, where the application of its EO at 0.02% (*V*/*V*) in milk inhibits its presence for at least 15 days [79]. At the same concentration, it eliminates the presence of *Escherichia coli* after 3 days and with an MIC of 0.62 mg·mL and 0.60 mg·mL it inhibits *Listeria monocytogenes* and *Staphylococcus aureus* respectively [80]. The latter was eliminated after 7 days at a dose of 0.01% (*V*/*V*) of EO in milk [86] or, at a dose of 0.02% (*V*/*V*), with a reduction of −10^7^ CFU·g^−1^ after three hours [79].

The inhibition of *Clostridium* spp. has been crucial in preventing the late blowing defect in cheese, a process characterized by the formation of gases within the cheese mass, originating from this microorganism, which causes padding, cracks, irregular holes, and metabolites that can compromise both the cheese texture and sensory quality [87,88]. However, some strains have different resistance: *Clostridium tyrobutyricum* only exhibits generalized inhibition with oregano EE (*Origanum vulgare* L.), between 1.88 and 7.5 mg·mL^−1^, while *Clostridium beijerinckii* proves to be the most sensitive strain, showing inhibition with the essential oils of the following plants: oregano (*Origanum vulgare* L.), MIC: 0.06 mg·mL^−1^; Savory (*Satureja montana* L.), MIC: 0.12 mg·mL^−1^; Hyssop (*Hyssopus officinalis* L.), MIC: 0.06 mg·mL^−1^; Spanish Lavender (*Lavandula stoechas* L.) MIC: 0.31 mg·mL^−1^; Marjoram (*Origanum manjerona* L.), MIC: 0.04 mg/mL and tarragon (*Artemisia dracunculus* L.), MIC: 0.04 mg·mL^−1^ [81]. The studies carried out by the same author with EE showed greater efficacy than the OEs of the same plants: the tarragon (*Artemisia dracunculus*) EE proved to be the most effective against *Clostridium tyrobutyricum* (MIC: 11.7 μL·mL^−1^), *Clostridium butyricum* (MIC: 15.6 μL·mL^−1^) and *Clostridium sporogenes* (MIC: 7.8 μL·mL^−1^). EE Hyssop (*Hyssopus officinalis* L.) proved to be the most effective against *Clostridium beijerinckii* (MIC: 0.9 μL·mL^−1^), and EE de Spanish Lavender (*Lavandula stoechas* L.) against *Clostridium sporogenes* (MIC: 7.8 μL·mL^−1^). After 14 days of ripening, no clostridial spores were detected in the cheeses treated with hyssop, lavender and tarragon EE [81].

The remaining data compiled in Table 3 demonstrate the antimicrobial potential of the use of plants and their applicability to the dairy industry.

### 4.2. Nutritional, Sensorial, and Functional Properties Improvement Potential

Increased concern over the possible harmful effects of synthetic preservatives such as butylated hydroxyanisole (BHA), butylated hydroxytoluene (BHT), nitrates, sulphites, sorbates, and benzoates has led to the search for natural alternatives for cheese preservatives. Plant extracts, rich in phenolic compounds and antioxidants, can not only increase the stability and safety of cheeses, but also improve their nutritional and functional properties [89,90,91,92]. However, the inclusion of plant extracts and EO can influence the sensory profile of cheese, altering aroma, flavor and texture. Rosemary, oregano and thyme compounds, for example, contribute enjoyable herbal notes to certain cheese varieties, although an excess of volatile molecules can impair product acceptability. To overcome this limitation, encapsulation technologies have been explored to manage the release of bioactive compounds, avoiding losses by oxidation, volatilization, and degradation, and restricting unwanted interactions with the food matrix [91,93]. These compounds may be applied directly on the product or through active packaging and edible coatings, promising strategies for delaying lipid oxidation, shelf life extension, and reducing moisture and mass loss during storage. These approaches not only guarantee the microbiological stability of the cheese but also help maintaining its organoleptic properties and overall quality, making it an innovative and attractive solution for the dairy industry [89,93].

## 5. Microbiological Approaches

**Figure 2:**
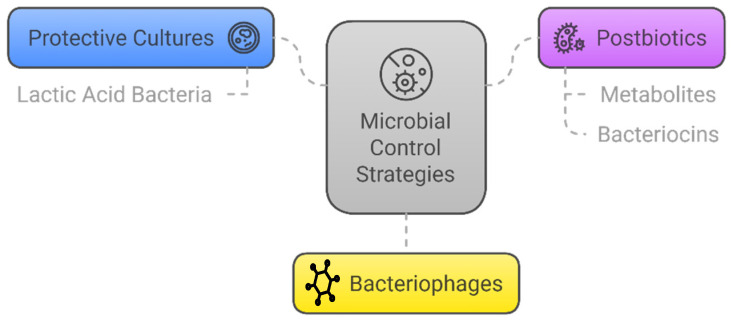
Microbial control strategies through microbiology: protective cultures, postbiotics, and bacteriophages.

The microbiological approach could be a way of effectively controlling the presence of unwanted microorganisms in cheeses. The diagram in Figure 2 summarizes this approach in its three ways: protective cultures, postbiotics, and bacteriophages.

### 5.1. Protective Cultures

LAB such as *Lactococcus*, *Streptococcus* and *Lactobacillus* are known to contribute to the characteristics of ripening cheeses and their quality, in sensory and physic-chemical, due to their role in enhancing the proteolysis and lipolysis that they develop [94]. These bacteria, naturally present in raw milk and added to pasteurized milk, are referred to as starter cultures. Besides the fermentation process of the cheese, they also provide a protective function [95,96].

These bacteria have been isolated, identified, and studied for their protective potential against a range of undesirable microorganisms. Table 4 shows some of the most recent work in this area.

The analysis of the data in Table 4 shows that several studies have investigated the effects on *Listeria monocytogenes*, yielding variable results. A reduction effect has been observed due to the action of *Lactococcus lactis*, *Lactiplantibacillus plantarum*, *Bifidobacterium breve*, and *Bifidobacterium animalis* [97,98,101,103,107]. A protective effect has been associated with *Lactobacillus delbrueckii* ssp. *sunkii* [96,99], while an inhibitory effect has been particularly noted in ripened cheese containing *Enterococcus faecium* CRL1879, *Lacticaseibacillus casei* 116, and *Lacticaseibacillus garvieae* 151 [100,107].

Previous studies on the action of LAB against Shigatoxin-producing *Escherichia coli* (STEC) revealed that a combination of *Hafnia alvei*, *Lactiplantibacillus plantarum* and *Lactococcus lactis* exhibited strong inhibitory action, reducing STEC O26:H11 and O157:H7 by more than 3 log CFU·g^−1^ [108].

Nevertheless, not all types of cheese show positive results, particularly fresh cheeses where LAB tend not to inhibit the growth of *Listeria monocytogenes*, highlighting the importance of ripening time for LAB activity [109]. Makki et al. [110] investigated this approach in cottage cheese, using commercial formulations containing *Lacticaseibacillus* spp., *Lactiplantibacillus* spp., and *Lacticaseibacillus rhamnosus*. These cultures effectively delayed the growth of molds (*Aspergillus cibarius*, *Penicillium citrinum*, *Penicillium roqueforti*, *Mucor genevensis*, *Mucor racemosus*) and yeasts (*Debaryomyces hansenii*, *Meyerozyma guilliermondii*, and *Torulaspora delbrueckii*). However, this study warns against the ineffectiveness of these protective cultures on some microorganism characteristics of cheese, such as those of *Candida zeylanoides* and *Penicillium commune*.

The LAB are essential not only for technological purposes in the production of cheeses with characteristic organoleptic parameters and consumer acceptance, but also in food safety. The addition of LAB enhances food safety, especially when it comes to cheeses produced with raw milk, preventing outbreaks of infections such as listeriosis, and infections caused by *Escherichia coli*, among other potentially lethal diseases. However, its action is conditioned by a few factors such as the initial level of contamination, ripening conditions, (temperature, humidity, ripening time), and overall production hygiene [95]. It is important to highlight, as noted by Rendueles et al. [111], that LAB has the potential to stop or delay the onset of future contamination and not to control situations in which the initial concentrations of contaminants are high. The action of LAB makes it possible to suppress other inhibition processes that rely on additives or synthetic antimicrobials, thus making cheese a Clean Label product.

### 5.2. Postbiotics

The action of LAB was analyzed to understand how their presence affects microorganisms and what biological mechanisms are involved. Based on these studies it was possible to realize the presence of a set of postbiotics, bioactive components produced by microorganisms which, in the context of cheese production, can be used to control undesirable microorganisms [112]; in this case, they can be divided into two types: metabolites and bacteriocins.

#### 5.2.1. Metabolites

The metabolites produced by LAB in cheeses play a fundamental role in cheese preservation. One of the main sets of metabolites are organic acids, such as acetic, lactic and propionic acids, among others, which, due to the decrease pH and consequent acidification of the environment, lead to a destabilization process of the plasma membrane, altering gradients and stopping transmembrane transport. In addition to these acids, there are other metabolites such as hydrogen peroxide, fatty acids (e.g., decanoic, choriolic) and reactive oxygen species, all of which are a set of compounds that make microbiological survival and proliferation impossible. This highlights the importance of carefully selecting and studying LAB strains to optimize their protective effects [94,113,114].

#### 5.2.2. Bacteriocins

Bacteriocins, peptides, or ribosomal proteins are naturally produced by LAB with antimicrobial properties. There are several isolated bacteriocins identified with potential use in cheeses, such as lacticin 3147 and 481, macedocin, nisin, pediocin, reuterin, and thermophilin 110 [115,116,117]. Table 5 summarizes the inhibitory effects of some of these compounds on undesirable microorganisms.

Nisin, produced by *Lactococcus lactis*, turns out to be the only one approved for use as a food additive, E234 [19], not only in ripened cheeses but also for other food products.

However, there have been some difficulties in the mass production of these compounds, either due to the complexity of production, isolation and purification, the associated economic cost, or their resistance to the proteolytic enzymes used during coagulation and ripening, which affect the stability of these bacteriocins [95].

The postbiotics released by the microorganisms (enzymes, vitamins, short chain fatty acids, exopolysaccharides, neurotransmitters, extracellular vesicles, etc.) have yet to be evaluated in their pharmacological functions as antioxidant, antineoplastic, anti-diabetic, and anti-inflammatory agents thus having a positive potential for the consumer [123].

### 5.3. Bacteriophages

Bacteriophages are specific viruses that act on microorganisms and are still seen as harmful elements in the cheese production process. Their destructive and inhibitory actions on fermentative bacteria jeopardize production, impairing the quality of final products and resulting in major losses for producers [124]. However, the biological advantage of these phages has been sought with their use in reducing and controlling the incidence of harmful microorganisms in the cheese; their specificity and harmlessness to humans have led bacteriophages to be studied, over the last few years, as biocontrollers of a whole range of microorganisms present in cheese [125,126].

The study by García-Anaya et al. [127] demonstrated the effectiveness of bacteriophage A511 against *Listeria monocytogenes* when applied to the surface of cheese using a whey protein isolate-based coating, achieving a reduction of 0.86 log CFU·g^−1^. Meanwhile, Komora et al. [128] successfully eliminated the microorganism, when inoculated at levels of 10^4^ CFU·mL^−1^, in combination with HPP.

Additionally, the control of *Clostridium tyrobutyricum*, responsible for blowing defects, was achieved using the FA67 phage, which delayed the defect by 14 days during ripening [129].

*Staphylococcus aureus* was able to be attacked with the phages KMSPI and phiPLA-RODI, whether through direct application to the milk, the surface of sliced cheese, or incorporation into coating [130,131]. The total elimination of *Pseudomonas mosselii* was achieved using phages ΦC106 and Φ21A, and the action on *Pseudomonas* is an area where further studies will be carried out to prevent surface defects characteristic of these microorganisms, namely pigmentation [132].

Table 6 summarizes and provides more information carried out in recent years.

In 2006, EFSA issued a positive scientific opinion on the use of the bacteriophage *PhageGuard Listex™ P100* for controlling *Listeria monocytogenes* in certain ready-to-eat foods, including meat and poultry, fish and shellfish, and dairy products. However, EFSA emphasized that this bacteriophage should be applied only as an additional measure to enhance microbial food safety, in conjunction with good hygienic and manufacturing practices, rather than as a replacement for these essential practices [133].

Following on from the studies of bacteriophages, new lines of research have emerged, particularly into endolyssin, or lysins, enzymes produced by bacteriophages responsible for breaking down peptidoglycan walls, which have a high degree of specificity and efficiency; one example is the endolyssin LysFA67, which causes the rapid lysis of *Clostridium tyrobutyricum* [134], the only limitation of these enzymes being their action on Gram-positive bacteria, which have peptidoglycan walls.

Bacteriophages can also be used to produce specific disinfectants and cleaning solutions when there is environmental contamination in cheese production facilities, especially in equipment and ripening chambers. However, as already mentioned, this approach can have negative aspects when, inadvertently, a phage is introduced into an industrial plant that, due to its characteristics or evolutionary processes, can lead to the deregulation of the lactoflora that are beneficial to the fermentation process of cheese and the organoleptic characteristics of the final product [135,136,137].

The microbiological options presented will be entirely valid from a Clean Label perspective, however, they may raise concerns for less informed consumers, potentially making them to perceive this approach as less natural. This perspective of this approach could be minimized by a communication and consumer information plan.

## 6. By-Products

The concept of a circular economy linked to the primary sector, where the reuse of waste and its valorization has been promoted both in the economic and political spheres. From these products, previously seen as waste, compounds have been obtained with potential in the field of natural food additives, such as polysaccharides, organic acids, and proteins, being a way of replacing additives with options that increase the consumer’s perception of Clean Label and an opportunity to valorize and give potential to agro-industrial by-products [138,139].

There are many examples in this field, namely extracts obtained from tomato by-products [140,141], grape pomace [142,143], olive pomace, and many others. Taking olive pomace as an example, the extracts contain bioactive compounds such as phenolic compounds including flavonoids and hydroxytyrosol, as well as alpha-tocopherol (Vitamin E) and fatty acids. These compounds have demonstrated the ability to inhibit the growth of Gram-positive bacteria (*Staphylococcus aureus*), Gram-negative bacteria (*Escherichia coli*) and fungi (*Candida albicans*) [144]. Additionally, olive pomace extract is considered safe for consumption [145].

### By-Products of the Dairy Industry

Cheese production can have a significant environmental impact, and whey, the main by-product of this production, can be recovered and valorized.

In general, the production of 1 kg of cheese generates between 9 and 10 L of whey; this whey can also be used to produce whey cheese and curd cheese, leading to the generation of a second-whey by-product. Both have a high Biochemical Oxygen Demand (BOD) and a high Chemical Oxygen Demand (COD) which, when disposed of in water courses or sewage systems, puts the dynamics of ecosystems at risk, especially in the case of this second whey with BOD = 50 g·L^−1^ and COD 80 g·L^−1^ which has no valid industrial use [146].

The valorization of this second whey, in addition to having a positive impact on the environment, will encourage economic and product circularity and it could have a significant impact on the application of Clean Label.

The protein content of the second whey can vary from between 6 and 8 g·L^−1^ [147,148]. The enzymatic or microbiological action on these proteins leads to the formation of peptides that have shown significant antimicrobial potential.

The study of these peptides with antimicrobial properties has been an attractive area of study [148,149]. Milk, the raw material for cheese, has been a source of this type of organic molecule, which in many cases crosses paths with cheese production.

**Figure 3:**
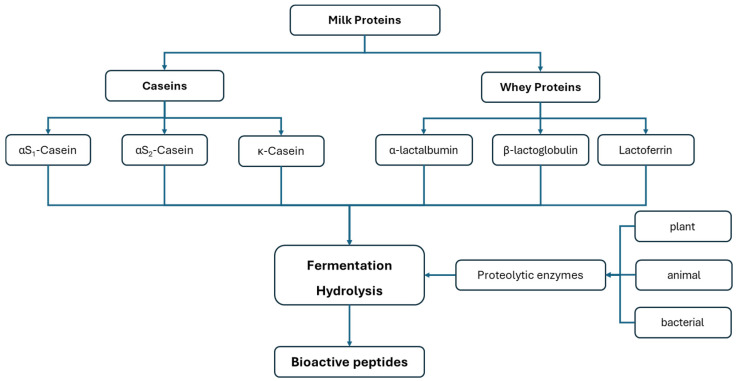
Biosynthetic pathways of bioactive peptides in the dairy industry.

The diagram in Figure 3 illustrates the potential pathways for producing peptides with bioactive potential.

The hydrolysis of αS2-Casein, by the action of chymosin (the enzyme responsible for coagulating milk when rennet is used) leads to the formation of the peptide casocidin, which shows microbial activity against *Staphylococcus* spp., *Sarcina* spp., *Bacillus subtilis*, *Diplococcus pneumoniae*, and *Streptococcus pyogenes*. The action of this peptide on both Gram-positive and Gram-negative is verified with doses between 8 and 95 µmol·mL^−1^ [150].

The action of chymosin on αS1-Casein also produces isracidin, a peptide with antimicrobial capacity against *Candida album* and *Staphylococcus aureus*, in the latter with an efficacy of 80% [151].

Lactoferrin, a glycoprotein, is another whey component, which has broad antimicrobial activity, whether against Gram-negative and positive bacteria, fungi, or viruses [152].

The action of pepsin on lactoferrin produces lactoferricin, which demonstrates full effectiveness in controlling spoilage of *Pseudomonas* spp. [153]. LAB also contribute significantly to the production of these peptides with antimicrobial properties, an example being Caseicin’s A, B, and C, which have an antibiotic capacity against *Escherichia coli* O157:H7 and *Cronobacter sakazakii*, in addition to the aforementioned bacteria [154].

Many of the peptides obtained also have important pharmacological properties: by hydrolyzing α-lactalbumin- and β-lactoglobulin-used enzymes obtained from *Cynara cardunculus* L. (a plant used in the coagulation of traditional cheeses in Portugal and Spain [155]), two peptides are produced with an inhibitory function on angiotensin converting enzyme are obtained, thus having anti-hypertensive properties by inhibiting angiotensin converting enzymes (ACE) [149,156].

The action of the enzymes Alcalase and Corollase on β-lactoglobulin produces a set of four peptides with antioxidant properties [157], while trypsin produces peptides with antimicrobial properties [158].

We must also consider that obtaining permeates with a high mineral content through ultrafiltration or the reverse osmosis process, which is essential for isolating whey fractions, we can produce a set of food additives that can be reintegrated into cheese production.

The use of these peptides and whey fractions thus eliminates the need for preservatives that aim to reduce the activity of microorganisms harmful to humans, maintaining cheese safety in a Clean Label environment. Furthermore, this approach adds value to compounds and by-products of cheese manufacture that would otherwise be discarded.

## 7. Natural and Edible Coatings

Edible films and coatings are an emerging alternative to conventional plastics, in that they have the unique advantage of being consumed with the food product, without any need for removal. Such materials contribute not only to environmental sustainability and are produced with industrial by-products, but also add to the stabilization of food by extending its shelf life and maintaining its structural integrity. Moreover, these materials are biodegradable, renewable, and mostly nontoxic, hence being an environmentally friendly option with little environmental impact [159,160].

Many by-products of the food industry are being studied to produce biopolymers, whey being one of most promising. Edible whey film is a highly interactive, dry polymeric network with a three-dimensional gel-like structure. According to the production techniques, the final films may present a spatial rearranged gel matrix incorporating additional film-forming agents. These films are usually produced by the casting method, where the solution is spread on a flat surface to form a dry gel, which may be used as a coating for food products. In the case of coatings, food products are dipped in the film-forming solution for about 30–60 s to ensure complete surface coverage, good adhesion, and structural integrity [161,162,163].

Whey-based films and coatings are characterized by their flexibility, transparency, colorlessness, and lack of odor. Unique properties such as amphiphilic behavior, electrostatic charges, charge density, and hydrophilic–hydrophobic balance influence their conformation, ultimately determining the physical and mechanical characteristics of the films and coatings [164].

These materials exhibit favorable physical properties at low relative humidity, including oxygen impermeability and excellent aroma retention. However, they have a high-water vapor permeability because of their hydrophilic nature. This weakness can be compensated by the addition of hydrophobic agents such as essential oils or plant extracts that increase the content of hydrophobic groups, reducing water vapor permeability and can also add to the antimicrobial properties mentioned previously [165]. Other advantages of whey-based films include their water solubility and the ease of preparation with water as the film-forming solvent. Their transparency, flexibility, and neutral sensory properties—odorless and tasteless—further increase their acceptance by consumers [159,164].

Incorporating bioactive compounds into whey-based films, such essential oils, microorganisms like LAB [166,167], probiotics [168] or postbiotics [169,170], could provide coating with new antioxidants, antibacterials and antifungals properties, thereby extending the cheese’s shelf life.

Another innovative approach in the field of biofilms involves using alternative animal by-products, such as sheep’s wool [171]. Traditionally used in textiles, wool is a source of keratin, a structural protein with properties for developing biopolymers. Keratin-based biopolymers have won attention due to their biodegradability, film-forming capacity and potential functional applications, particularly in the food and packaging industries. Wool has become a product with less and less commercial value, and approximately 12 million tons of wool are discarded worldwide every year. Efficient and sustainable extraction methods, such as enzymatic hydrolysis or green chemistry approaches, could improve the use of keratin in biodegradable films and coatings, offering an ecological alternative to synthetic polymers and simultaneously responding to the challenges of waste recovery, circular economy, and Clean Label [172,173].

## 8. Clean Label as a Health Promoter

The Clean Label ideal is based on promoting consumer health and wellbeing by eliminating or reducing substances that are perceived or scientifically proven to compromise health. It also reflects a growing consumer trend towards more natural and less processed foods with recognizable ingredients [174].

Throughout this article, we have discussed various approaches that can contribute to this goal, such as the suppression of synthetic antimicrobials, the elimination of artificial additives unrelated to the cheese’s natural matrix, and the reduction of salt content, a traditional preservative whose excessive consumption is directly related to cardiovascular problems [175,176,177]. The Clean Label concept can therefore have a broadly positive impact on public health by promoting safer and healthier alternatives.

Besides these strategies, clean labelling opens the door to innovations in the dairy sector, developing new proposals such as adding bioactive and functional compounds for health benefits. In this regard, some strategies that could be used to protect cheese from lipid oxidation are based on antioxidant molecules obtained from plant extracts, improving its stability but, at the same time, possibly contributing to consumer health. Similarly, fortification with vitamin D_3_, by exposing the cheese to UV light, can make up for some of the nutritional deficiencies common in the population [58,59].

Recovery and reutilization of mineral whey fractions, generally undervalued, may reintroduce these important nutrients into the process chain, enhancing the sustainability and nutritional value of cheeses. Furthermore, the addition of bioactive peptides with pharmacological properties and prebiotics will enhance not only the functionality of the product but also have a direct positive impact on the intestinal microbiota and health of consumers [178,179], as well as some of these biopeptides also having potential antihypertensive and antidiabetic action [180,181]. It is also worth mentioning some studies that demonstrate the action of LAB in reducing cholesterol levels and in modelling the fat profile of cheese [182].

Finally, Clean Label must also guarantee the microbiological safety of cheeses, maintaining strict standards of quality and control during the manufacturing process.

## 9. Challenges, Risks and Opportunities

### 9.1. Food Safety and Conservation

Reducing or eliminating the use of standard additives and preservatives, as well as making changes to production processes, must not distract from the important focus on ensuring food safety [183].

Between 2018 and 2022, EFSA [184] reported that milk and dairy products were linked to 149 confirmed outbreaks in Europe, resulting in 1754 human cases, 298 hospitalizations, and 22 fatalities. *Salmonella* spp. was the most prevalent pathogen, accounting for 27.52% of these outbreaks. *Staphylococcus aureus* toxins were implicated in 15.44%, and Shiga toxin-producing *Escherichia coli* (STEC) was identified in 6.04%. Although *Listeria monocytogenes* was associated with only 4.03% of the outbreaks, it was responsible for most fatalities, causing 14 out of the 22 reported deaths. Listeriosis is a major concern in the United States, with outbreaks linked to the consumption of dairy products, particularly cheeses. Production and packaging factors have been reported as contributors to these contaminations [185]. This was followed by *Streptococcus zooepidemicus*, which, despite being linked to only one outbreak, resulted in five fatalities [184].

Because of this data, food safety is something that a producer should never neglect when developing and placing a Clean Label cheese on the market.

### 9.2. Sensorial Quality

The sensory properties of cheese can be affected by the use of natural preservatives, though the extent of this impact depends on the specific preservative, its concentration, and the type of cheese. Sensory properties like flavor, texture, aroma, and appearance are often altered, either positively or negatively, by preservatives—natural or synthetic. Regarding the flavor, natural preservatives such as rosemary extract may add a subtle herbal or pine-like flavor, whereas a citrus extract might lend a slightly sour or tangy note. In texture, some natural preservatives, like essential oils or plant extracts, can alter the texture by affecting the microbial balance or moisture retention. Finally, the aroma: natural preservatives can influence the microbial community by limiting spoilage organisms, which may change the overall aroma profile [77,93,186]. The appearance can also be impacted by some natural preservatives (for example rosemary extract) altering the color slightly, although this fact can also occur using artificial preservatives which could impact consumer acceptance [93].

### 9.3. Shelf-Life

The shelf life of cheese varies depending on its type, moisture content, and storage conditions. Generally, hard cheeses (e.g., cheddar, parmesan) have longer shelf lives compared to soft cheeses (e.g., ricotta, brie), due to their lower moisture content and more acidic pH, which inhibits bacterial growth. For many cheeses, shelf life ranges from a few weeks to several months (even up to a year for hard cheeses), but it also depends on the presence of preservatives and the conditions during transportation and storage [187,188].

In the cheese industry, Clean Label technologies aim to extend the shelf life of products without using artificial preservatives and many of these strategies have already been explained in this article: reducing the harmful microbial content; using preservatives obtained from natural sources, such as plants; or using biologically sustainable packaging that enhances the preservation of cheese [77,189,190].

### 9.4. SWOT Analysis

**Figure 4:**
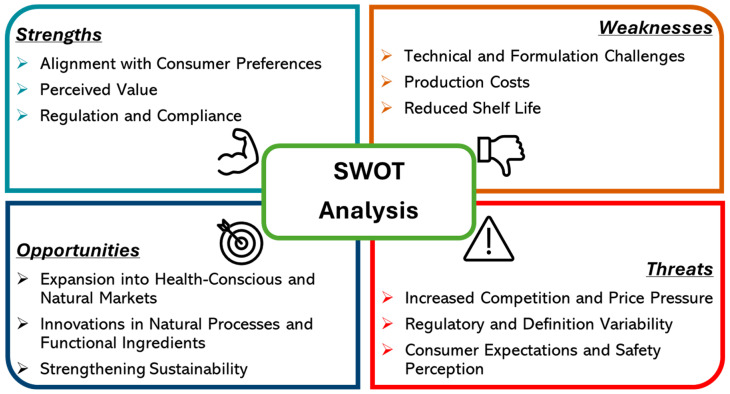
SWOT analysis of the application of the clean label to cheese from an intersectoral point of view.

In addition to the factors already mentioned, there are several variables that can significantly influence the applicability of the Clean Label concept in the dairy industry, not only from a technological standpoint, but also from an economic, regulatory and cross-sectoral point of view. Given this background, a SWOT (Strengths, Weaknesses, Opportunities and Threats) analysis was carried out with the aim of identifying the main factors influencing the implementation of Clean Label in the dairy industry and providing a strategic vision for companies intending to adopt this concept. This analysis is an essential step towards understanding the challenges and opportunities associated with the transition to more sustainable practices in line with modern consumer expectations. Figure 4 aims to summarize the main aspects of this analysis.

Strengths: Clean Label promises to resonate with a rising group of consumers who opt to be more conscious and attentive to simpler food ingredients. Clean Label products mean an overall growing demand for natural and simple ingredients through either a drastic reduction or the elimination of synthetics and artificial additives. This will reassure consumers, build brand loyalty, and foster repeat business [191,192,193]. These trends have allowed cheese makers to position themselves as champions of the heal-conscious and wholesome marketplace, aligning with consumer expectations for integrity in product formulation. Finally, Clean Label products, with their premium perception, often support price points that compete in the organic, natural, and artisanal cheese segments [194,195]. This allows producers to provide added quality to their products, something that is highly recognized in a market that favors sustainable production and traditional production methods [189,196]. The elimination of artificial additives facilitates compliance with food safety mandates, and dovetails perfectly with strict international standards, like those in the EU where purity of ingredients is heavily regulated. Such a regulatory harmonization could ease market access for Clean Label cheese in areas where safety requirements are stringent and provide a boost to global distribution [193].

Weaknesses: Clean Label improvements generally involve processes which include the removal of stabilizers, preservatives, and emulsifiers which, in the case of manufactured cheese products, can bring about complications such as decreased stability, textural attributes, and shelf-life [192]. Such additives play a critical role, especially in ripened cheeses where precise metabolic pathways are practiced; to refrain from including them may lead to sub-optimal product quality which will warrant the modification of substantial technologies and inventive modern manufacturing methodologies. It should be noted that the search for natural substitutes for these chemical ingredients makes manufacturing more expensive and less productive [197,198]. For a small or medium-sized producer, the effective modification of recipes to change the product to a more Clean Label version requires substantial investment in research and development, which is expensive [199]. Additionally, the lack of artificial preservatives usually translates to shorter durability in products which challenges logistics and distribution. This limitation can narrow the potential market, especially for export markets with high expectations of speed in transportation or excessive requirements for refrigeration which would increase the cost of distribution [198].

Opportunities: Growing natural and health-aligned products offers massive channels through which Clean Label cheese expands health-conscious markets, especially the ones in the US and Western Europe. First and foremost, Clean Label cheese attracts not only those with health consciousness and/or orientation towards sustainability, but even a consumer with different reasons, such as dietary restrictions and allergies or other environmental issues. Thus, Clean Label becomes part of the niche market in pocketing consumer willingness to pay more for high-quality cheese products [200,201]. This trend is further supported by the advancements of biotechnology in developing natural microbial cultures and plant-derived ingredients to serve as alternatives to traditional additives. In addition, advances in active packaging that aim to prolong the shelf life of food products without synthetic preservatives have great potential for the distribution of Clean Label cheese, especially in regions with strict food safety and food purity requirements [202]. The Clean Label is closely associated with sustainable production as it focuses on local and organic ingredients [203]. Furthermore, environmental commitments could also help reinforce the appeal of a Clean Label product environmental certificate while enhancing brand reputation in the sustainability-focused market segment [204].

Threats: The demand for Clean Label makes competition very aggressive in both artisanal and natural cheese categories. Larger companies have more resources to execute manufacture at lower costs using Clean Label strategies. Thus, the competitive set of companies makes it difficult for a small production unit to keep prices competitive without hurting their margins [205]. Also, the term “Clean Label” without a meaning is going to mislead consumers and will allow for “greenwashing”: a process in which manufacturers describe their business as “green” and, hence, lure more customers into the market [206]. Moving the goalposts, the rate at which modifications take place in food safety regulations, may also mean that Clean Label formulations are modified periodically, adding to operational costs and product marketing complexity [10]. Moreover, such high quality and durable expectations by consumers might not be realized without additives. Clean Label cheeses, at this moment, have shorter shelf lives or changed textural and flavor profiles compared to those with preservatives, and this may lead to dissatisfaction among consumers, especially in countries where refrigeration may be limited [16].

## 10. Conclusions

There has been huge interest in the Clean Label movement from the cheese industry in recent years, inspired by consumer interest in more natural and less processed food without synthetic additives. The major technological, microbiological, and botanical strategies presently under evaluation for Clean Label compliance while ensuring food safety, sensory quality, and shelf life have been discussed. Non-thermal alternatives for microbial control include physical approaches like HPP, PEF, and VL, which are promising for maintaining the nutritional and functional properties of cheese. Similarly, microbiological approaches, such as the application of LAB protective cultures, postbiotics, and bacteriophages, also show potential to replace conventional preservatives with naturally occurring antimicrobial agents. Edible coatings and botanical extracts have been among the most important solutions in the extension of shelf-life and functional improvement of cheese products in recent years as part of Clean Label formulation.

Despite this progress, important obstacles persist. Typically, reformulations to Clean Label status demand the removal of some key ingredients like stabilizers, preservatives, and emulsifiers, often with negative effects on texture and consistency and shortened shelf life. Therefore, more research should be directed toward the development of novel methods for preserving more stable Clean Label cheese with undegraded quality. Research on emerging antimicrobial and antioxidant compounds, particularly the use of biopeptides obtained directly from the dairy industry. Packaging and coating represent an innovative approach, either using by-products such as whey or through more technologically advanced solutions such as polymers derived from sheep’s wool. As well as promoting environmental circularity in the sector, these strategies valorize by-products, generate economic and environmental benefits, and reinforce the perception of Clean Label in the dairy industry.

The development of more robust methods for implementing the Clean Label, as well as the exploration of synergies between different approaches that can be applied in combination, are areas where research is still insufficient and needs to be further developed. Another crucial aspect to consider, which has become evident throughout this review, is the gap in the transfer of knowledge, methodologies, and technologies to the industry. No matter how much progress research makes, if it is not effectively disseminated and applied by producers, its impact and relevance become limited.

The SWOT analysis of Clean Label in cheese has made it possible to understand the interaction between the various players in the dairy industry, as well as the regulatory and socio-economic factors involved. This study revealed not only the potential of this approach but, even more clearly, the threats and challenges that could impact the sector.

After all, Clean Label cheese is not just a trend but a movement of change in the quest for health, sustainability, and transparency in the dairy industry. Further exploration of innovative, science-based solutions will continue to enable the industry to meet the rising demand for more natural and high-quality cheeses while ensuring food safety and regulatory compliance.

## Figures and Tables

**Table 1:** Effect of HPP (pressure, time and temperature) on undesirable microorganisms in milk.

Target	Treatment	Effect	Ref.
*Escherichia coli*	600 MPa, 5 min, 40 °C	−3.00 log CFU·mL^−1^	[36]
	600 MPa, 5 min, 25 °C	−6.80 log CFU·mL^−1^	[37]
	250 MPa, 10 min, 25 °C	−6.39 log CFU·mL^−1^	[33]
*Listeria monocytogenes*	600 MPa, 10 min, 25 °C	−5.91 log CFU·mL^−1^	[37]
*Pseudomonas* spp.	600 MPa, 3 min, 25 °C	Total elimination	[37]
*Staphylococcus aureus*	600 MPa, 25 min, 25 °C	−4.70 log CFU·mL^−1^	[38]
Strain: *765*	345 MPa, 5 min, 50 °C	>−8.0 CFU·mL^−1^	[39]

CFU: Colony-forming unit.

**Table 2:** Inhibitory effect on undesirable microorganisms of PL, UV and VL in milk and cheese (fresh and ripened).

Target	Matrix	Treatment	Effect	Ref.
*Enterobacteriaceae*	Mozzarella fresh	PL, 7.02 J·cm^−2^, 2 s	Total elimination	[49]
	Fresh ricotta	PL, 1.03 J·cm^−2^, 5 s	Delaying spoilage	[46]
*Escherichia coli*	Milk	VL, 413 nm, 720 J·cm^−2^, 2 h	>−5 log	[51]
Strain: O157:H7	Fresh kashar	UV, 100–11,000 nm, 45 s	−3.02 log CFU·cm^−2^	[52]
*Listeria* spp.	Gouda, slice	PL, 0.90 J·cm^−2^, 5 s	−3 log CFU·cm^−2^	[50]
		PL, 12.0 J·cm^−2^, 3 pulses·s^−1^, 360 µs	−3.37 log CFU·cm^−2^	[45]
*Listeria monocytogenes*	Sliced	UV, 222 and 307 nm, 80 s	−3.20 log CFU·g^−1^	[53]
	Packaged	VL, 460–470 nm, 4 d	Total elimination	[54]
*Pseudomonas* spp.	Fresh ricotta	PL, 3.10 J·cm^−2^, 5 s	Delaying spoilage	[46]
*Pseudomonas fluorescens*	Mozzarella fresh	PL, 7.02 J·cm^−2^, 2 s	Total elimination	[49]
	Packaged	VL, 460–470 nm, 4 d	Total elimination	[54]
*Pseudomonas aeruginosa*	Milk	VL, 413 nm, 720 J·cm^−2^, 2 h	>−5 log	[51]
*Salmonella typhimurium*	Sliced	UV, 222 and 307 nm, 80 s	−3.50 log CFU·g^−1^	[53]
	Milk	VL, 413 nm, 720 J·cm^−2^, 2 h	>−5 log	[51]
*Staphylococcus aureus*	Milk	VL, 413 nm, 720 J·cm^−2^, 2 h	>−5 log	[51]
	Fresh kashar	UV, 100–11,000 nm, 45 s	−1.62 log CFU·cm^−2^	[52]

CFU: Colony-forming unit; PL: Pulsed light; UV: Ultraviolet; VL: Visible light; Packaged: application in packaged cheeses; Sliced and slice: application in sliced cheeses.

**Table 3:** Antimicrobial effect of plant extracts.

Target	Botanical Species	1	2	3	Dose	Result	Ref.
*Aspergillus flavus*	Oregano (*Origanum vulgare* L.)	EO	milk	R	0.02% (*V*/*V*)	Inhibited (15 days)	[79]
*Bacillus cereus*	Basil (*Ocimum basilicum* L.)	EO	curd	F	MIC: 0.08 mg·mL^−1^		[80]
	Marjoram (*Origanum manjerona* L.)	EO	curd	F	MIC: 0.31 mg·mL^−1^		[80]
	Oregano (*Origanum vulgare* L.)	EO	curd	F	MIC: 0.08 mg·mL^−1^		[80]
*Clostridium* spp.	Oregano (*Origanum vulgare* L.)	EE	milk	R	MIC: 35.2 μL·mL^−1^		[81]
		EO	milk	R	MIC: 0.06 mg·mL^−1^		[81]
*Clostridium beijerinckii*	Marjoram (*Origanum majorana* L.)	EO	milk	R	MIC: 0.04 mg·mL^−1^		[81]
	Savory (*Satureja montana* L.)	EO	milk	R	MIC: 0.12 mg·mL^−1^		[81]
*Clostridium. tyrobutyricum*	Hyssop (*Hyssopus officinalis* L.)	EE	milk	R	MIC: 23.4 μL·mL^−1^		[81]
	Spanish Lavender (*Lavandula stoechas* L.)	EE	milk	R	MIC: 31.3 μL·mL^−1^		[81]
	Marjoram (*Origanum majorana* L.)	EE	milk	R	MIC: 125 μL·mL^−1^		[81]
	Savory (*Satureja montana* L.)	EE	milk	R	MIC: 11.7.6 μL·mL^−1^		[81]
	Tarragon (*Artemisia dracunculus* L.)	EE	milk	R	MIC: 15.6 μL·mL^−1^		[81]
*Escherichia coli*	Oregano (*Origanum vulgare* L.)	EO	milk	R	0.02% (*V*/*V*)	Elimination after 3 days	[80]
	Fennel (*Foeniculum vulgare* Mill.)	D	curd	F	MIC: 1.00 mg·mL^−1^		[82]
	Marjoram (*Origanum majorana* L.)	EO	curd	R	MIC: 1.25 mg·mL^−1^		[80]
	Basil (*Ocimum basilicum* L.)	EO	curd	F	MIC: 0.075 mg·mL^−1^		[80]
*Listeria monocytogenes*	Myrtle (*Myrtus communis* L.)	EO	milk	R	MIC: 31.25 μL·mL^−1^	1–2 log CFU/g reduction relative to control	[83]
	Basil (*Ocimum basilicum* L.)	EO	curd	F	MIC: 1.25 mg·mL^−1^		[80]
	Rosemary (*Rosmarinus officinalis* L.)	EO	milk	R	MIC: 0.40 μL·mL^−1^	1–2 log CFU/g reduction relative to control	[83]
	Oregano (*Origanum vulgare* L.)	EO	curd	F	MIC: 0.62 mg·mL^−1^		[80]
	Marjoram (*Origanum majorana* L.)	EO	curd	F	MIC: 2.5 mg·mL^−1^		[80]
*Penicillium* sp.	Caraway (*Carum carvi* L.)	EO	closed atmosphere	R	MIC: 0.250 μL·mL^−1^		[84]
	Fennel (*Foeniculum vulgare* Mill.)	D	curd	F	MIC: 0.40 mg·mL^−1^		[82]
	Litsea (*Litsea cubeba* Lour. Per.)	EO	closed atmosphere	R	MIC: 0.016 μL·mL^−1^		[84]
	Marjoram (*Origanum majorana* L.)	EO	closed atmosphere	R	MIC: 0.250 μL·mL^−1^		[84]
	Thyme (*Thymus vulgaris* L.)	EO	closed atmosphere	R	MIC: 0.063 μL·mL^−1^		[84]
	Red thyme (*Thymus serpyllum* L.)	EO	closed atmosphere	R	MIC: 0.125 μL·mL^−1^		[84]
*Penicillium verrucosum*	Thyme (*Thymus vulgaris* L.)	EO	milk	R	0.025 mg·g^−1^	Total inhibition (4 months)	[85]
*Staphylococcus aureus*	Oregano (*Origanum vulgare* L.)	EO	milk	R	0.02% (*V*/*V*)	−10^7^ CFU·g^−1^ after 3 h	[79]
		EO	curd	F	MIC: 0.60 mg·mL^−1^		[80]
		EO	milk	F	0.01% (*V*/*V*)	Total elimination after 7 days	[86]
	Ginger (*Zingiber officinale* Roscoe)	EO	milk	F	0.01% (*V*/*V*)	Total elimination after 14 days	[86]
	Basil (*Ocimum basilicum* L.)	EO	curd	R	MIC: 0.075 mg·mL^−1^		[80]
	Marjoram (*Origanum manjerona* L.)	EO	curd	F	MIC: 1.25 mg·mL^−1^		[80]

1: Extract type; D: Decoction; EE: Ethanolic extracts; EO: Essential oil; 2: Method of application; 3: Cheese type: F: Fresh; R: Ripened cheese; Closed atmosphere: Cheese packaged in a closed atmosphere system without gas or moisture exchange; MIC: Minimum inhibitory concentration.

**Table 4:** Action of protective cultures (LAB) on undesirable microorganisms in cheese.

Target	Protective Culture	Action	Ref.
*Listeria monocytogenes*	*Lactococcus lactis*, *Lactiplantibacillus plantarum*	Lowered counts by 3–4 log units compared to the control	[97]
	*Companilactobacillus crustorum*; *Lactiplantibacillus plantarum*; *Limosilactobacillus fermentum*	−0.52 log units units compared to the control	[98]
	*Lactobacillus delbruekki* ssp. *sunkii*	−0.3–1.8 log CFU·g^−1^, non-detectable after 90 days of ripening	[99]
	*Enterococcus faecium* CRL1879	Undetectable up to 30 days of ripening with initial inoculation of 10^3^ CFU·mL^−1^ without organoleptic changes	[100]
	*Lactococcus lactis* CAU2013	Reduce the growth by 1 log unit	[101]
	*Lacticaseibacillus casei* 116; *Lactococcus garvieae* 151	−3.57 log CFU·g^−1^ after 90 days of ripening	[102]
	*Bifidobacterium breve*; *Bifidobacterium animalis*	−10.29 log CFU·g^−1^ after 21 days	[103]
*Bacillus cereus*	*Lacticaseibacillus paracasei*	Decrease the counts in Kareish cheese	[104]
	*Lactiplantibacillus plantarum*	In vitro IZD: 3.2 ± 0.61 mm	[105]
*Clostridium tyrobutyricum*	*Lactococcus lactis* spp. *lactis 32*	−0.6 log units units compared to the control	[106]

IZD: Inhibitory Zone Diameter.

**Table 5:** Inhibitory effects of some bacteriocins on undesirable microorganisms.

Target	Bacteriocin	Action	Ref.
*Listeria ivanovii*	Pediocin PA-1	MIC: 0.09 μg·mL^−1^	[118]
	Reuterin	MIC: 250 μg·mL^−1^	
	Nisin	MIC: 1.56 μg·mL^−1^	
*Listeria monocytogenes*	Pediocin	−2 log, 30 d, 4 °C	[119]
		>−4 log CFU·mL^−1^	[116]
	Thermophilin 110	≥640 AU·mL^−1^, inhibited growth	[116]
	Lacticin 3147	MIC: 0.99 μg·mL^−1^	[120]
	Reuterin	150 units·g^−1^, 3 d, −4.8 log CFU·mL^−1^	[121]
*Clostridium tyrobutyricum*	Reuterin	MIC: 4.06 mM	[122]
	Nisin	MIC: 6.25 μg·mL^−1^	[122]
*Salmonella enterica*	Reuterin	MIC: 125 μg·mL^−1^	[118]
*Escherichia coli* O157:H7	Reuterin	150 units·g^−1^, 7 d, undetectable	[121]

MIC: Minimum inhibitory concentration; AU: Activity Units.

**Table 6:** Bacteriophage activity against undesirable microorganisms in cheese.

Target	Phage	Action	Note	Ref.
*Listeria monocytogenes*	A511	Bacterial counts reduced 0.86 log CFU·g^−1^	In a whey protein isolate-based edible coating	[127]
	P100	Eliminated when inoculated with levels of 10^4^ CFU·mL^−1^	Combined effect with HPP	[128]
*Clostridium tyrobutyricum*	FA67	Late blowing defect on day 14 of ripening		[129]
*Staphylococcus aureus*	KMSP1	−8.8 CFU·mL^−1^ in milk −4.3 CFU·cm^−2^ in sliced cheddar		[130]
	phiIPLA-RODI	Reduction and control	Gelatine films remained	[131]
*Pseudomonas mosselii*	ΦC106 Φ21A	Total elimination in milk		[132]

## Data Availability

No new data were created or analyzed in this study. Data sharing is not applicable to this article.
